# Survival Outcomes of Patients with Treated vs Actively Surveilled Bosniak III or IV Kidney Cysts: A Systematic Review

**DOI:** 10.1016/j.euros.2026.05.007

**Published:** 2026-06-01

**Authors:** Carla Chehadeh, Vikas Burugu, Paul E. Andrews, Alyssa K. McGary, Heidi E. Kosiorek, Matthew D.F. McInnes, Eric Lam, Lisa A. Marks, Nelly Tan

**Affiliations:** a Mayo Clinic Alix School of Medicine, Mayo Clinic College of Medicine and Science, Phoenix, AZ, USA; b University of Texas Medical Branch, John Sealy School of Medicine, Galveston, TX, USA; c Department of Urology, Mayo Clinic, Phoenix, AZ, USA; d Department of Quantitative Health Sciences, Mayo Clinic, Scottsdale, Arizona, USA; e Department of Radiology, University of Ottawa, Ottawa, ON, Canada; f Methodological and Implementation Research, Ottawa Hospital Research Institute, Ottawa, ON, Canada; g Library Services, Mayo Clinic, Scottsdale, AZ, USA; h Department of Radiology, Mayo Clinic, Scottsdale, AZ, USA

**Keywords:** Active surveillance, Bosniak classification, Bosniak III, Bosniak IV, Cancer-specific survival, Kidney cystic masses, Overall survival, Renal cell carcinoma

## Abstract

**Background and objective:**

Bosniak III and IV kidney cysts have high malignancy rates, and treatment is often recommended. However, evidence suggests active surveillance (AS) may achieve similar cancer-specific survival (CSS) and overall survival (OS). We systematically reviewed studies comparing AS and treatment outcomes for these cysts.

**Methods:**

We searched Ovid MEDLINE, Ovid Embase, Scopus, and Web of Science to identify studies in English reporting CSS or OS outcomes for patients with Bosniak III or IV kidney cysts. Risk of bias was assessed with the Quality in Prognosis Studies tool. The Grading of Recommendations Assessment, Development, and Evaluation approach was used to provide a certainty of evidence summary. Per-study estimates were reported.

**Key findings and limitations:**

Five retrospective studies met the inclusion criteria. Across studies of Bosniak III cysts, 5-yr CSS was consistently >98% in both treated and AS cohorts, with several studies reporting no cancer-specific deaths among cases managed with AS. Studies of Bosniak IV cysts also had 5-yr CSS that exceeded 93% for both treatment strategies, including cohorts with 100% CSS in selected AS patients. OS was reported inconsistently and tended to be lower in AS cohorts with Bosniak III cysts, reflecting differences in baseline age, comorbidity burden, and competing causes of mortality rather than true differences in oncologic treatment. All studies were retrospective and nonrandomized, with moderate to high levels of selection bias and confounding.

**Conclusion and clinical implications:**

AS may be an appropriate management strategy for carefully selected patients with Bosniak III and IV cysts.

## Take Home Message

Active surveillance in patients with Bosniak III and IV kidney cysts was associated with high observed 5-yr cancer-specific survival rates and may represent a potential management option for carefully selected patients, warranting further investigation.

ADVANCING PRACTICE **What does this study add?**

To our knowledge, this is the first systematic review to compare cancer-specific survival (CSS) and overall survival between treated and actively surveilled patients with Bosniak III or IV kidney cysts. Our findings show that active surveillance yielded excellent survival outcomes, with 5-yr CSS exceeding 98% across active surveillance groups. Our findings challenge the assumption that all complex cysts require immediate intervention and provide support for individualized patient management. These findings are susceptible to selection bias and study heterogeneity and require further investigation.

**Clinical relevance**

This systematic review suggests that carefully selected patients with Bosniak III or IV kidney cysts managed with active surveillance experience very high observed 5-year cancer-specific survival, challenging the assumption that all complex cysts require immediate intervention. While the available evidence is limited to retrospective, nonrandomized studies with potential selection bias and confounding, these findings support individualized risk-adapted decision-making and highlight the need for prospective comparative studies. Associate Editor: Maria Carmen Mir.

**Patient summary**

We compared the percentage of patients with complex (ie, Bosniak III or IV) kidney cysts who survived their cancer 5 yr after being either surgically treated or actively surveilled. We found that the percentage of patients who survived their cancer at 5 yr was higher than 93% for both groups. This suggests that some carefully selected patients with kidney cysts may not need immediate surgery and can be closely monitored in the appropriate clinical context.

## 1 Introduction

Cystic lesions of the kidney are common, with a prevalence that increases with age [1]. A high percentage of kidney cysts are discovered incidentally during evaluation for unrelated conditions [2]. The Bosniak classification system is widely used for categorizing kidney cysts according to their risk for malignancy on the basis of imaging characteristics [3]. The classification spectrum ranges from Bosniak I, which describes benign, simple cysts, to Bosniak IV, which indicates high suspicion for malignancy. Thus, malignancy rates are highest for Bosniak III (50–80%) [4] and IV (85–90%) cysts, posing implications for their management [5], [6].

The American Urological Association (AUA) recommends that the management of Bosniak III and IV kidney cysts be guided by the estimated malignancy risk, the patient’s comorbid conditions, and patient preferences. However, recent studies have shown that complex cystic kidney masses, particularly Bosniak III and IV cystic lesions, often exhibit indolent tumor biology and can have favorable outcomes with active surveillance (AS). Notably, AS involves regular monitoring and follow-up to detect changes that may require management and does not indicate a lack of treatment. In appropriately selected patients, especially those with limited life expectancy or high surgical risk, AS with repeat imaging in 3–6 mo is a viable strategy for assessing interval growth or radiographic changes that may warrant delayed intervention. For patients with longer life expectancy and acceptable surgical risk, the standard of care for Bosniak IV lesions is minimally invasive partial or radical nephrectomy. For Bosniak III lesions, either surgery or AS may be considered. The AUA emphasizes individualized, risk-adapted decision-making with shared discussion between the clinician and patient.

Despite their higher malignancy rates, Bosniak III and IV cysts tend to not be aggressive and often have low grades on pathologic evaluation [7]. The indolent nature of these cysts indicates higher survival rates and suggests the possibility that they are overtreated. Overtreatment may lead to unnecessary partial or radical nephrectomies, which carry risks of bleeding, infection, injury to adjacent organs, anesthetic complications, and chronic kidney disease [8]. A systematic review showed that malignancy risk in Bosniak III cysts may be overestimated, supporting the rationale for AS over immediate surgery [9]. Analysis of cost effectiveness further supports AS as the preferred strategy for managing Bosniak III kidney cysts, with surgery being reserved for cystic masses of increased size or worrisome features [10].

Studies comparing prognostic outcomes between treated vs AS cohorts are limited. Although some studies suggest a substantial proportion of lesions are benign or display indolent behavior, most data are derived from retrospective surgical cohorts with variable levels of bias. In this systematic review, we aimed to evaluate 5-yr prognostic outcomes for patients with Bosniak III and IV cysts who underwent AS or treatment with ablation or surgery.

## 2 Methods

### 2.1 Literature search

This systematic review was registered through PROSPERO (Prospective Register of Systematic Reviews; ID: CRD42024618417). A literature search was conducted in the following databases: Ovid MEDLINE, Ovid Embase, Scopus, and Web of Science (Appendix). The database searches were completed by using a combination of keywords and MeSH (medical subject headings) terms. Keywords included *kidney cancer*; *renal cell carcinoma*; *cystic renal cell carcinoma*; *cystic kidney cancer*; cystic kidney disease*; *active surveillance*; *ablation*; *cryoablation*; *surgery*; *partial nephrectomy*; *interventional radiology*; *Bosniak classification*; *Bosniak* 2*f*; *Bosniak 3*; *Bosniak 4*; *Bosniak IIf*; *Bosniak III*; *survival*; *mortality*; and *hazard ratio*. MeSH terms included *Kidney Neoplasms*; *Carcinoma*, *Renal Cell*; *Kidney Diseases*, *Cystic*; *Watchful Waiting*; *Ablation Techniques*; *Radiofrequency Ablation*; *General Surgery*; *Nephrectomy*; *Radiology*, *Interventional*; *Survival*; and *Mortality*. The search was limited to articles published in English.

### 2.2 Study identification and selection

Two reviewers (C.C. and V.B.) independently selected studies for inclusion in the systematic review by using Covidence (Veritas Health Innovation Ltd). Titles and abstracts were initially screened, and studies that were not relevant to the topic of interest were excluded. For the remaining studies, full-text articles were screened by using inclusion and exclusion criteria. Inclusion criteria included analysis of Bosniak III or IV kidney cysts, reporting of overall survival (OS) and cancer-specific survival (CSS) as clinical outcomes, human studies, publication in English, patient age of 18 yr or older, and availability of the full-text article. Exclusion criteria included non-English publications, abstracts and conference papers, studies involving patients under 18 yr of age, preclinical animal studies, and studies reporting outcomes other than OS and CSS. Disagreements were resolved by deliberation between the 2 reviewers. If agreement was not reached, a third reviewer (N.T.) was consulted.

### 2.3 Data collection

The 2 reviewers (C.C. and V.B.) independently extracted information from the included studies by using standardized data extraction forms created specifically for this systematic review. Extracted data included study year, authors, country of study, sample size, median sample age, median sample gender distribution, follow-up time period, subgroup sample size (Bosniak III, Bosniak IV, treated, AS), treatment status (ablation, surgery, or AS), clinical outcomes (CSS, OS), definition of outcome, hazard ratio (if available), Kaplan-Meier curve (if available), patient selection process, selection bias, type of study, imaging modality for kidney mass classification, conflicts of interest, funding, and reported study limitations. For studies that had missing data, we contacted the corresponding author to request additional data. For studies that did not report percentages, the reviewers calculated percentages by using the available numbers. When studies explicitly reported the number of cancer-specific deaths and the corresponding cohort size, CSS was calculated as a simple proportion. For studies that reported percentages describing management groups, these percentages were used to estimate subgroup sample sizes when absolute counts were not provided. When survival outcomes were reported only as percentages or Kaplan-Meier estimates, these values were extracted and reported as published. No event counts or time-to-event data were reconstructed from Kaplan-Meier–derived survival estimates. For studies that had sex distribution data and follow-up times split by Bosniak classification, the average was calculated to report the overall sex distribution, and the median was reported for follow-up time. All outcomes were summarized descriptively at the individual study level; no pooled estimates or meta-analytic aggregation was performed. When studies reported lesion-level data, we assessed whether lesion counts corresponded directly to unique patients. Lesion-level percentages were converted to patient counts only when a 1-to-1 correspondence between lesions and patients was explicitly stated or when patient-level outcomes were directly reported. When such correspondence could not be confirmed, lesion-level data were reported descriptively and were not combined with patient-level survival outcomes.

### 2.4 Quality assessment

The Cochrane Prognosis Methods Group recommends using the Quality In Prognosis Studies (QUIPS) tool to assess risk of bias in prognostic studies [11]. The QUIPS tool assesses the following 6 relevant domains when evaluating the quality of evidence for prognostic studies: study participation, study attrition, prognostic factor measurement, outcome measurement, study confounding, and statistical analysis and reporting [12]. The 2 reviewers (C.C. and V.B.) independently conducted a quality assessment for each study by using the QUIPS tool to assess bias. After independent review, the 2 reviewers discussed the findings to resolve any disagreements. If disagreement remained, a third reviewer (N.T.) was consulted.

The Grading of Recommendations Assessment, Development, and Evaluation (GRADE) approach was used to provide a certainty of evidence summary. GRADE is a systematic framework for assessing the certainty (or quality) of evidence in systematic reviews. The GRADE system evaluates evidence across multiple domains including risk of bias (study design limitations), consistency (variability in results between studies), precision (adequacy of sample size and confidence intervals), directness (applicability to the question of interest), and publication bias [13], [14].

### 2.5 Data analysis

For the purpose of a meta-analysis, studies were examined for hazard ratios and Kaplan-Meier survival curves to assess the viability of a pooled estimate of survival data (in the absence of hazard ratios). A formal meta-analysis of the survival outcomes was not possible due to the lack of reported hazard ratios in most studies, insufficient data to reconstruct individual patient data from Kaplan-Meier curves, the absence of numbers at risk at multiple time points, and missing information on censoring patterns. Similarly, confidence intervals could not be calculated and pooled analyses could not be performed due to unavailability of the necessary data. A narrative synthesis and summary of the available data was performed.

## 3 Results

### 3.1 Study selection

**Fig. 1:**
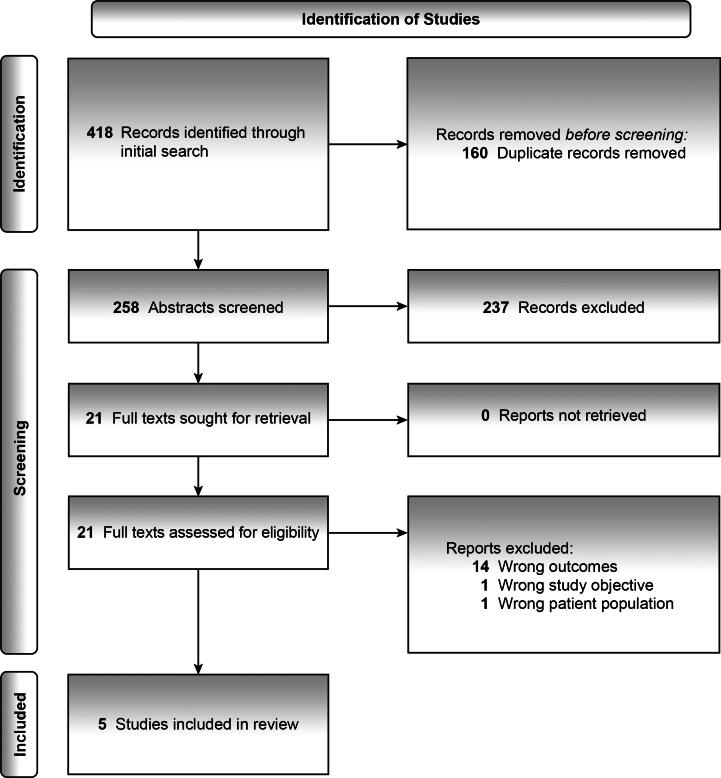
PRISMA (Preferred Reporting Items for Systematic Reviews and Meta-Analyses) flow diagram of the study selection process.

Our initial search resulted in 418 references (Fig. 1). After duplicates were removed (*n* = 160), a total of 258 citations underwent title and abstract review. Studies were excluded that mentioned Bosniak III or IV renal cysts without evaluating survival outcomes, or that were review articles, meta-analyses, or other nonoriginal study types. Only original clinical studies were included. A total of 237 records were excluded during title and abstract review. Of the 21 studies that proceeded to full-text review, 5 were included (Table 1, Table 2 [15], [16], [17], [18], [19]). All 5 of the studies were retrospective analyses with study periods ranging from 2000 to 2020. The studies originated from multiple countries including Canada, France, Finland, and the United States. Non-English language publications were excluded due to feasibility constraints related to translation and data extraction. This restriction was applied uniformly during study screening and selection. Patients were categorized according to whether they underwent AS or treatment (ie, ablation or surgery) for Bosniak III or IV kidney cysts (Table 3). A total of 579 patients had Bosniak III cysts (AS, *n* = 246; treated, *n* = 333), and 393 patients had Bosniak IV cysts (AS, *n* = 74; treated, *n* = 319). Boissier et al. [16] reported no patients monitored with AS. Median ages spanned 56–63.5 yr. The proportion of male participants ranged from 56% to 69%. Median follow-up periods ranged from 61–67.5 mo (Table 1). Kidney masses were classified by using computed tomography (CT), magnetic resonance imaging (MRI), and/or ultrasonography (US). However, Boissier et al. [16] and Lee et al. [18] did not specify the imaging modalities used. Chandrasekar et al. [15] and Luomala et al. [17] explicitly used CT, MRI, or US. Kashan et al. [19] used CT or US. Notably, none of the studies referenced the 2019 updated Bosniak classification.

**Table 1:** Characteristics of the Included Studies^a^ (*n* = 5)

**Authors and reference**	**Publication year**	**Country of origin**	**Study type**	**Imaging modality for kidney mass classification**	**Time frame for patient cases**	**Overall median follow-up time (mo)**	**Bosniak III median follow-up time (mo)**	**Bosniak IV median follow-up time (mo)**	**Median age (yr)**	**Men (%)**
Chandrasekar et al. [15]	2018	Canada	Retrospective	CT, MRI, US	2001–2003	65	65	65	58	60
Boissier et al. [16]	2019	France	Retrospective	NR	2000–2010	65	65	65	56	69
Luomala et al. [17]	2022	Finland	Retrospective	CT, MRI, US	2006–2017	NR	62	73	64	59
Lee et al. [18]	2023	United States	Retrospective	NR	2000–2020	NR	NR	61	62	63
Kashan et al. [19]	2018	United States	Retrospective	CT, US	2000–2015	65	NR	NR	61	56

CT = computed tomography; MRI = magnetic resonance imaging; NR = not reported; US = ultrasonography.

a Some values were rounded to the nearest whole number for consistency of presentation.

**Table 2:** Summary of Tumor Size, AS Conversion Rate, and Histopathologic Findings Across Studies (*n* = 5)

**Study**	**Tumor size**	**Rate of switch to AS**	**Histopathologic details of treated patients**
Chandrasekar et al. [15]	Median (IQR): Bosniak III, 3.5 (2.3–5.7) cm; Bosniak IV, 3.8 (2.3–8.6) cm	No upgrades from Bosniak III to IV	Intervention group: clear cell RCC (44.6%), multilocular cystic RCC (20.0%), tubulocystic RCC (1.5%), papillary type 1 RCC (15.4%), papillary type 2 RCC (3.1%), chromophobe RCC (1.5%), benign pathology (4.6%), sarcoma (1.5%), unknown/ablation (7.7%)
Boissier et al. [16]	Mean (SD): Bosniak III, 4.9 (3.5) cm; Bosniak IV, 5.9 (4.5) cm^a^	All patients treated	Bosniak III: conventional (58%), chromophobe (2%), papillary (23%), cystic multilocular clear cell (13%), cystic conventional (4%) Bosniak IV: conventional (62%), chromophobe (5%), papillary (26%), cystic multilocular clear cell (7%), cystic conventional (0%)
Luomala et al. [17]	Median (IQR): Bosniak III, 2.9 (2.0–5.1) cm; Bosniak IV, 4.3 (2.8–6.1) cm	Bosniak III: 7.1% upgraded to IV; median time to intervention, 12 mo Bosniak IV: median time to intervention, 19 mo	Bosniak III: clear cell RCC (*n* = 35), papillary RCC (*n* = 11), unspecified (*n* = 4), multilocular cystic RCC (*n* = 6), chromophobe RCC (*n* = 3), translocation carcinoma (*n* = 1) Bosniak IV: clear cell RCC (*n* = 63), papillary RCC (*n* = 21), unspecified (*n* = 8), multilocular cystic RCC (*n* = 2), chromophobe RCC (*n* = 2), translocation carcinoma (*n* = 0)
Lee et al. [18]	Median (IQR): AS, 2.2 (1.5–3.4) cm; immediate intervention, 3.4 (2.2–5.5) cm	Delayed intervention: Bosniak III, 21%; Bosniak IV, 29%	Histopathology not reported
Kashan et al. [19]	Median (IQR): AS, 3.1 (2.3–4.5) cm; intervention, 3.3 (2.2–4.7) cm	28.9% switched from AS to surgery	Surgery group: clear cell RCC (65.9%), multilocular cystic renal cell neoplasm of low malignant potential (13%), benign multilocular cyst (11.6%), papillary RCC (3.6%), clear cell capillary RCC (2.9%), unclassified RCC (1.4%), mixed epithelial and stromal tumor family (1.4%)

AS = active surveillance; RCC = renal cell carcinoma.

a The original study reports these values as 49 (35) cm and 59 (45) cm, but this is likely a unit reporting error.

**Table 3:** Summary of 5-Yr Survival Outcomes for Studies of Bosniak III and IV Kidney Cysts a, b

**Study**	**Bosniak III**				**Bosniak IV**			
	**5-yr CSS (%)**		**5-yr OS (%)**		**5-yr CSS (%)**		**5-yr OS (%)**	
	**Treated**	**AS**	**Treated**	**AS**	**Treated**	**AS**	**Treated**	**AS**
Chandrasekar et al. [15]	100 (*n* = 37)	100 (*n* = 85)	NR	NR	94 (*n* = 18)	100 (*n* = 11)	NR	100 (*n* = 11)
Boissier et al. [16]	NR	NR	95 (*n* = 81)	NR	NR	NR	96 (*n* = 62)	NR
Luomala et al. [17]	100 (*n* = 54)	99 (*n* = 85)	NR	NR	99 (*n* = 85)	100 (*n* = 21)	NR	NR
Lee et al. [18]	100 (*n* = 91)	100 (*n* = 64)	96 (*n* = 91)	83 (*n* = 64)	94 (*n* = 95)	100 (*n* = 33)	83 (*n* = 95)	85 (*n* = 33)
Kashan et al. [19]	100 (*n* = 70)	100 (*n* = 12)	NR	NR	100 (*n* = 59)	100 (*n* = 9)	NR	NR

AS = active surveillance; CSS = cancer-specific survival; NR = not reported; OS = overall survival.

a The n values in parentheses indicate the number of patients in each group.

b Some values were rounded to the nearest whole number for consistency of presentation.

None of the studies reported hazard ratios. However, Kaplan-Meier survival curves were presented in 3 studies (Chandrasekar et al. [15], Boissier et al. [16], and Lee et al. [18]). Two studies (Luomala et al. [17] and Kashan et al. [19]) mentioned Kaplan-Meier curves but did not provide them.

### 3.2 CSS and OS for patients with Bosniak III kidney cysts

CSS for patients with Bosniak III cysts was consistently high across all included studies, with 5-yr estimates exceeding 98% regardless of management strategy (Table 3). Both treated and AS cohorts showed excellent long-term cancer-specific outcomes, with no clear directional signal suggesting inferior cancer control among cases managed with AS compared with treatment. These findings support the interpretation that, in selected patients, deferring immediate intervention is not associated with worse cancer-specific outcomes within the reported follow-up periods of approximately 5–6 yr.

In contrast, among patients with Bosniak III cysts, OS was lower in AS cohorts than in treated cohorts, with those studies reporting absolute differences of more than 10 percentage points (Table 3). This pattern is most likely attributable to differences in baseline patient characteristics rather than differences in oncologic outcomes. Patients selected for AS were more likely to be older and have a higher burden of comorbid conditions, increasing the confluence of competing risks for noncancer mortality. The limited and inconsistent reporting of OS across studies further hinders interpretation and underscores the effect of selection bias inherent to retrospective observational cohorts.

### 3.3 CSS and OS for patients with Bosniak IV kidney cysts

Among patients with Bosniak IV kidney cysts, 5-yr CSS exceeded 93% across both treated and AS cohorts, with multiple studies reporting 5-yr CSS near or at 100% in selected AS populations (Table 3). Despite the increased risk of malignancy associated with Bosniak IV lesions, no consistent pattern of worse cancer-specific outcomes was observed among selected cases managed with AS. These findings suggest that, in carefully selected patients, AS was associated with favorable cancer-specific outcomes, within the limits of observational data.

OS outcomes for patients with Bosniak IV cysts were broadly similar between AS and treated cohorts in the limited number of studies reporting this end point (Table 3). In contrast to CSS, which remained consistently high across management strategies, OS was more variable, most likely reflecting differences in baseline patient health rather than differences in oncologic control. Clinically, this distinction underscores the importance of individualized decision-making that weighs patients’ comorbid conditions, life expectancy, and competing risks alongside the potential benefits of immediate intervention.

### 3.4 Methodologic quality assessment of included studies With QUIPS

Results of the risk of bias assessment performed by using the QUIPS tool are summarized in Table 4. All studies were judged to have a moderate risk of bias in the domain of study participation. Because these were nonrandomized studies, treatment group assignment was subject to selection bias. This weakens the comparability between groups and may overstate the apparent benefits of treatment, especially when OS is used instead of CSS. Study attrition was rated as high risk in 4 studies and as moderate risk in 1. This was due to limited follow-up reporting and lack of longitudinal patient data, which introduced uncertainty in outcome completeness. All studies demonstrated low risk in prognostic factor measurement, reflecting consistent and reliable definitions of the prognostic variables relevant for this investigation. Outcome measurement was categorized as low risk in 3 studies and as moderate risk in 2, suggesting some variation in how outcomes were defined or assessed. Study confounding bias risk was found to be moderate in 1 study and high in 4 studies, indicating an increased risk of confounding factors affecting the outcomes. Statistical analysis and presentation were judged to be low risk in 4 studies and moderate in 1, reflecting largely appropriate analytical approaches and adequate reporting.

**Table 4:** QUIPS Bias Assessment^a^

**Study**	**Study participation**	**Study attrition**	**Prognostic factor measurement**	**Outcome measurement**	**Study confounding**	**Statistical analysis and presentation**
Chandrasekar et al. [15]	Moderate	Moderate	Low	Low	Moderate	Low
Boissier et al. [16]	Moderate	High	Low	Moderate	High	Low
Luomala et al. [17]	Moderate	High	Low	Low	High	Low
Lee et al. [18]	Moderate	High	Low	Moderate	High	Low
Kashan et al. [19]	Moderate	High	Low	Low	High	Moderate

QUIPS = Quality in Prognosis Studies.

a Studies are judged to have high, moderate, or low risk of bias.

Overall, the methodologic quality assessment of the included studies with QUIPS suggests acceptable reliability in measurement and analysis, despite limitations arising from moderate risk of bias in study participation due to nonrandomization, potential for unadjusted confounding related to treatment group selection, and incomplete follow-up data.

### 3.5 Certainty of evidence summary (GRADE narrative)

The overall certainty of evidence for 5-yr CSS and OS in patients with Bosniak III and IV kidney cysts managed with AS vs intervention is moderate.

#### 3.5.1 Risk of bias

All included studies are retrospective and nonrandomized, introducing a moderate risk of selection bias and confounding, particularly in treatment assignment and baseline patient characteristics. The QUIPS assessment identified moderate to high risk of bias in study participation, attrition, and confounding domains but low risk in outcome measurement and statistical analysis.

#### 3.5.2 Consistency

CSS was high in both groups (AS and treatment) across included studies; however, direct comparative inference is limited by confounding and the lack of adjusted estimates. This finding is robust and supported by large cohorts and guideline recommendations [20].

#### 3.5.3 Directness

The evidence directly addresses the population of interest (ie, patients with Bosniak III/IV cysts) and the critical outcomes (ie, CSS, OS) relevant to clinical decision-making. However, moderate heterogeneity exists in imaging modalities and outcome definitions.

#### 3.5.4 Precision

Sample sizes are moderate, and follow-up periods are adequate; however, the lack of randomized trials and limited reporting of hazard ratios or time-to-event data reduce precision. OS estimates may be confounded by comorbid conditions and selection factors.

#### 3.5.5 Publication bias

Evidence of publication bias was not assessable. The literature is limited to retrospective cohorts and may underrepresent negative or equivocal findings.

#### 3.5.6 Summary statement

Across the retrospective cohorts studied, CSS was consistently high among carefully selected patients who underwent AS. However, the certainty of comparative evidence between AS and intervention is limited by confounding, selection bias, and the absence of adjusted or study-specific comparative estimates. Accordingly, these findings should be interpreted as descriptive observations rather than as evidence of causal equivalence between management strategies. These findings are consistent with the cautious incorporation of AS into guideline discussions for selected patients and underscore the need for individualized decision-making and higher-quality comparative evidence [20].

## 4 Discussion

In this systematic review, we report 5-yr CSS and OS rates for patients with Bosniak III or IV kidney cysts who underwent AS vs treatment (ie, surgery or ablation). Across 5 retrospective studies, the 5-yr CSS exceeded 93% for all patients with Bosniak III or IV cysts, with a 5-yr CSS >98% for carefully selected patient groups undergoing AS. To our knowledge, this is the first systematic review of prognostic outcomes in patients with Bosniak III kidney cysts.

A recent systematic review and meta-analysis of kidney cysts classified with the 2019 Bosniak classification system showed that Bosniak III cysts have a high malignancy rate, with pooled proportions of malignancy reported to be around 80% (95% CI, 71–87) [4]. Despite this high malignancy rate, our study supports the notion that prognosis is excellent (5-yr CSS >98% for AS cohorts across studies, and 5-yr CSS rates of 100% for treated cohorts across studies) and that these cysts can be managed with AS rather than immediate treatment in the appropriate clinical context. Although OS was reported by only a select subset of studies, Lee et al. [18] notably reported that 5-yr OS was lower for patients undergoing AS (83%) than for treated patients (96%) (Table 3). This may have been attributed to patient factors such as age, comorbid conditions, or selection factors that influenced long-term outcomes. Alternatively, patients monitored with AS may have had other health conditions, worse baseline health, or a shorter life expectancy, resulting in patients and clinicians not pursuing surgical treatment. Despite patient factors, 5-yr overall CSS across studies was high. The current AUA guidelines suggest that AS with delayed intervention can be considered for patients with Bosniak III lesions [20]. Our findings suggest that AS was associated with high 5-yr CSS observed across studies of selected cohorts with Bosniak III and IV kidney cysts. Because all available data are derived from retrospective, nonrandomized cohorts with potential selection bias, confounding, and limited time-to-event reporting, these findings cannot establish the comparative effectiveness or safety of AS relative to immediate intervention.

The Bosniak classification system for cystic lesions of the kidneys is useful for predicting the risk of renal cell carcinoma, with the higher Bosniak classes carrying a higher risk of malignancy. For lesions classified with the 2019 Bosniak classification system, Bosniak III lesions have an 80% malignancy risk, whereas Bosniak IV lesions have an 88% malignancy risk [4]. The AUA guidelines recommend prioritizing intervention for Bosniak III and IV lesions, but AS may be considered when the risk of complications with intervention is greater than the benefit of decreasing the risk of malignancy [20], [21]. Percutaneous biopsy may be used as a risk stratification tool to distinguish benign lesions from malignant ones more accurately than imaging alone. Our results show that the survival prognosis for Bosniak III cysts is excellent, suggesting that biopsy may be an avenue to individualized management. Although the currently available radiographic classification systems are useful, they have various limitations in accurately distinguishing benign lesions from those that are malignant. In a study of 199 patients with Bosniak IIF and III complex kidney cysts, CT-guided biopsy led to a definitive diagnosis in 87.9% of cases, obviating the need for surgery or invasive procedures in up to 70% of patients with benign complex cysts [22].

We found that survival trends for Bosniak IV cysts were similar to those for Bosniak III cysts, though 5-yr CSS was slightly lower with treatment than with AS. The treatment group had a 5-yr CSS that ranged from 94% to 100%, whereas the AS group had a 100% 5-yr CSS in all relevant studies (Table 3). Lee et al. [18] reported a higher 5-yr CSS rate in the AS group than in the treatment group. This is surprising because treatment is generally expected to confer better cancer control through removal of the lesion. On one hand, patients chosen for AS may have had less aggressive tumors or more favorable imaging features (outside of the Bosniak classification). On the other hand, those selected for treatment may have had more suspicious or advanced lesions, leading to worse outcomes, despite intervention. Lee et al. [18] reported a higher rate of 5-yr OS in the AS group than in the treated group (85% vs 83%, respectively). These data indicate that, although Bosniak IV cysts have greater potential for malignancy than Bosniak III cysts, AS may still be an appropriate clinical management protocol, particularly for patients with increased surgical risk or lower life expectancy.

The high 5-yr CSS rate across treated and AS groups of patients with Bosniak III or IV cysts suggests that the natural progression of most cystic kidney malignancies is indolent, regardless of Bosniak class. AS was associated with excellent 5-yr CSS and OS rates for patients with Bosniak IV cysts, similar to patients with Bosniak III lesions, supporting that close monitoring can be considered even for Bosniak IV cystic kidney masses. Many of the conclusions drawn for Bosniak III cysts regarding the safety and viability of AS extend to Bosniak IV cysts. However, given that Bosniak IV lesions are associated with a higher malignancy rate (approximately 88%), treatment may be considered in a broader subset of patients. Although many Bosniak IV cysts may exhibit indolent behavior, the increased likelihood of high-grade malignancy or invasive features may justify treatment or close monitoring. The definitive histologic diagnosis and oncologic control provided by surgical or ablative interventions may make them preferable treatment options for these patients.

### 4.1 Limitations

This review is limited by selection bias, competing-risk mortality in the AS cohorts, and retrospective study design. In addition, patients who were monitored with AS may have had higher rates of comorbid conditions or a lower eligibility for surgery, resulting in confounded OS measurements. Furthermore, no studies reported hazard ratios or time-to-event outcomes, which limited the ability to assess differences in survival outcomes and pool estimates. These factors, in addition to the small cohort size of certain subgroups (eg, *n* = 74 across studies for Bosniak IV patients), limit the generalizability of our findings and warrant further investigation and careful patient selection for AS in the future. Given the small cohort sizes for certain subgroups, high-precision point estimates should be validated in future cohort studies with larger sample sizes.

A primary limitation of this review was the substantial heterogeneity in outcome classification and ascertainment across included studies. Sources of heterogeneity included variations in imaging modalities used to assign Bosniak classification, given that studies variably employed CT, MRI, or both. MRI may “upstage” cyst classification relative to CT, resulting in differences in classification and management strategies. Differences in outcome definitions also complicate interpretation. For instance, CSS may be defined strictly as the time from diagnosis to death directly attributed to renal cell carcinoma, whereas other studies may loosely classify any death as a cancer-related death in a patient with known malignancy and other comorbid conditions. Several studies did not specify the outcome definition used for reporting. Moreover, none of the studies included a centralized radiologic review process. Interpretation of complex kidney cysts is inherently subjective. Interobserver variation in applying the Bosniak classification system is well documented [23]. Lastly, some studies may have used earlier versions of the Bosniak classification system. Due to the revision of Bosniak criteria in 2019, the prognostic performance of previous classification systems may not translate to cohorts classified by using the 2019 system. This source of heterogeneity may affect the generalizability of this systematic review.

The data retrieval and calculations for our analysis also had limitations, given that the approximations performed during the data collection process are associated with potential errors. Some papers did not report percentages; therefore, the reviewers performed calculations from the available data. For papers that had only reported percentages, the numbers/sample sizes were calculated. In addition, not all papers reported the data that we aimed to analyze, and OS was reported in less than half the papers. For missing data, the reviewers attempted to contact study authors and were able to collect additional information.

Another limitation of this review was the exclusion of non-English language publications. Although this approach is common in systematic reviews, it may have resulted in incomplete capture of relevant international data and introduces the potential for language bias. This is particularly relevant given that management strategies and reporting practices for cystic renal lesions may vary across regions. Therefore, findings should be interpreted with appropriate caution when considering practice implications.

## 5 Conclusions

Our results support that patients with Bosniak III or IV kidney cysts have excellent CSS rates. Despite historically high malignancy rates, outcomes for patients in the AS group—particularly those with Bosniak III cysts—were found to be favorable, with 5-yr CSS rates close to 100% in carefully chosen patient populations. For patients with Bosniak IV cysts, similarly favorable outcomes observed between AS and treated groups suggest that AS may be considered in carefully selected patients, in addition to immediate intervention. Despite the limitations of this study, this is the first systematic review, to our knowledge, to show that AS is associated with positive outcomes depending on patient and clinical factors.

  ***Author contributions***: Nelly Tan had full access to all the data in the study and takes responsibility for the integrity of the data and the accuracy of the data analysis.

  *Study concept and design*: Chehadeh, Burugu, Tan.

*Acquisition of data*: Chehadeh, Burugu, Tan, Marks.

*Analysis and interpretation of data*: Chehadeh, Burugu, Tan, McGary, Kosiorek, Lam, McInnes.

*Drafting of the manuscript*: Chehadeh, Burugu, Tan, Marks.

*Critical revision of the manuscript for important intellectual content*: Chehadeh, Burugu, Tan, Andrews, McGary, Kosiorek, McInnes.

*Statistical analysis*: Chehadeh, Burugu, Tan, Marks, McGary, Kosiorek, Lam, McInnes.

*Obtaining funding*: None.

*Administrative, technical, or material support*: None.

*Supervision*: Tan.

*Other* (specify): None.

  ***Financial disclosures:*** Nelly Tan certifies that all conflicts of interest, including specific financial interests and relationships and affiliations relevant to the subject matter or materials discussed in the manuscript (eg, employment/affiliation, grants or funding, consultancies, honoraria, stock ownership or options, expert testimony, royalties, or patents filed, received, or pending), are the following: None.

  ***Funding/Support and role of the sponsor*:** None.

  ***Acknowledgments:*** The Scientific Publications staff at Mayo Clinic provided editorial consultation, proofreading, and administrative and clerical support.

  ***Data access and responsibility:*** Carla Chehadeh, Vikas Burugu, and Nelly Tan, MD, had full access to all the data in the study and take responsibility for the integrity of the data and the accuracy of the data analysis.

  ***Data sharing statement:*** All relevant data supporting the findings of this study are reported within the article or are available from the corresponding author upon reasonable request.

  ***Declaration of generative AI and AI-assisted technologies in the writing process:*** ChatGPT and OpenEvidence were used for initial idea generation and occasional editing throughout manuscript drafting. The authors reviewed and edited the content as needed and take full responsibility for the content of the publication.
