# Supplementary material for: Survival Outcomes of Patients with Treated vs Actively Surveilled Bosniak III or IV Kidney Cysts: A Systematic Review
Source: Eur Urol Open Sci. 2026 Jun 1;89:46–55. doi: 10.1016/j.euros.2026.05.007 (PMC13251640; doi:10.1016/j.euros.2026.05.007)
Supplement: Supplementary Data 1 [file mmc1.pdf]

Database(s): **Embase** 1988 to 2024 Week 27

Search Strategy:

#	Searches	Results
1	"kidney cancer".mp. or exp Kidney Neoplasms/	162227
2	"renal cell carcinoma".mp. or exp Carcinoma, Renal Cell/	86596
3	("cystic renal cell carcinoma" or "cystic kidney cancer").mp. [mp=title, abstract, heading word, drug trade name, original title, device manufacturer, drug manufacturer, device trade name, keyword heading word, floating subheading word, candidate term word]	514
4	1 or 2 or 3	166508
5	exp Kidney Diseases, Cystic/ or "cystic kidney disease*".mp.	21792
6	"active surveillance".mp. or exp Watchful Waiting/	24012
7	exp Radiofrequency Ablation/ or exp Ablation Techniques/ or ablation.mp.	221797
8	cryoablation.mp.	14161
9	surgery.mp. or exp General Surgery/	4236263
10	"partial nephrectomy".mp. or exp Nephrectomy/	77047
11	"interventional radiology".mp. or exp Radiology, Interventional/	34335
12	5 or 6 or 7 or 8 or 9 or 10 or 11	4446159
13	("bosniak classification" or "bosniak 2f" or "bosniak 3" or "bosniak 4" or "bosniak IIf" or "bosniak III" or "bosniak IV").mp. [mp=title, abstract, heading word, drug trade name, original title, device manufacturer, drug manufacturer, device trade name, keyword heading word, floating subheading word, candidate term word]	607
14	4 and 5 and 13	68
15	12 and 14	68
16	exp Survival/ or survival.mp.	2272203
17	mortality.mp. or exp Mortality/	1948163
18	"hazard ratio".mp.	229329
19	16 or 17 or 18	3842302
20	14 and 19	4
21	15 or 20	68
22	4 or 5	186399
23	12 and 22	86116
24	19 and 22	55951
25	23 or 24	116920
26	13 and 25	351
27	15 or 20 or 21 or 26	351

28	limit 27 to english language	320
29	limit 28 to (books or chapter or conference abstract or conference paper or "conference review")	136
30	28 not 29	184

1. Laparoscopic nephron-sparing surgery for complex renal cystic lesions: a single-center experience.

   Dong D., Zhang Y.

   Frontiers in Oncology. 14(no pagination), 2024. Article Number: 1398347. Date of Publication: 2024.

   [Article]

   Publisher
   Frontiers Media SA

   Cite

2. Update on Renal Cell Carcinoma Diagnosis with Novel Imaging Approaches.

   Bellin M.-F., Valente C., Bekdache O., Maxwell F., Balasa C., Savignac A., Meyrignac O.

   Cancers. 16(10) (no pagination), 2024. Article Number: 1926. Date of Publication: May 2024.

   [Review]

   Publisher
   Multidisciplinary Digital Publishing Institute (MDPI)

   Cite

3. Differentiating mixed epithelial and stromal tumor family from predominantly cystic renal cell carcinoma using magnetic resonance imaging-based Bosniak classification system version 2019.

   Guo H.-P., Xu W., Hao Y.-W., Kang H.-H., Zhang X.-J., Ding X.-H., Zhao J., Bai X., Zhou S.-P., Ye H.-Y., Wang H.-Y.

   Japanese Journal of Radiology. (no pagination), 2024. Date of Publication: 2024.

   [Article]

   Publisher
   Springer

Database: Ovid MEDLINE(R) 1946 to Present and Epub Ahead of Print, In-Process & Other Non-Indexed Citations and Ovid
MEDLINE(R) Daily
Search Strategy:
----
1 "kidney cancer".mp. or exp Kidney Neoplasms/ (89953)
2 "renal cell carcinoma".mp. or exp Carcinoma, Renal Cell/ (58028)
3 ("cystic renal cell carcinoma" or "cystic kidney cancer").mp. [mp=title, book title, abstract, original title, name of substance word, subject heading word, floating sub-heading word, keyword heading word, organism supplementary concept word, protocol supplementary concept word, rare disease supplementary concept word, unique identifier, synonyms, population supplementary concept word, anatomy supplementary concept word] (325)
4 1 or 2 or 3 (101861)
5 exp Kidney Diseases, Cystic/ or "cystic kidney disease*".mp. (17913)
6 "active surveillance".mp. or exp Watchful Waiting/ (14577)
7 ablation.mp. or exp Ablation Techniques/ or exp Radiofrequency Ablation/ (211577)
8 cryoablation.mp. (5150)
9 surgery.mp. or exp General Surgery/ (3183871)
10 exp Nephrectomy/ or "partial nephrectomy".mp. (40822)
11 "interventional radiology".mp. or exp Radiology, Interventional/ (13074)
12 6 or 7 or 8 or 9 or 10 or 11 (3313208)
13 ("bosniak classification" or "bosniak 2f" or "bosniak 3" or "bosniak 4" or "bosniak IIf" or "bosniak III" or
"bosniak IV").mp. [mp=title, book title, abstract, original title, name of substance word, subject heading word, floating sub-heading word, keyword heading word, organism supplementary concept word, protocol supplementary concept word, rare disease supplementary concept word, unique identifier, synonyms, population supplementary concept word, anatomy supplementary concept word] (348)
14 4 and 5 and 13 (141)
15 12 and 14 (64)
16 survival.mp. or exp Survival/ (1575316)
17 exp Mortality/ or mortality.mp. (1598032)
18 "hazard ratio".mp. (146600)
19 16 or 17 or 18 (2712770)
20 14 and 19 (13)
21 15 or 20 (66)
22 4 or 5 (116697)
23 12 and 22 (33570)
24 19 and 22 (25451)
25 23 or 24 (49472)
26 13 and 25 (131)
27 15 or 20 or 21 or 26 (131)
28 limit 27 to (english language and humans) (103)
